# Utilization of services among Korean, Vietnamese, and Non‐Hispanic White caregivers for persons with dementia/Alzheimer's disease

**DOI:** 10.1002/alz70858_107668

**Published:** 2025-12-26

**Authors:** Constance Emory‐Khenmy, Seunghye Hong, Jung‐Ah Lee

**Affiliations:** ^1^ University of Hawaii at Manoa, Honolulu, HI, USA; ^2^ University of California, Irvine, Irvine, CA, USA

## Abstract

**Background:**

The world's population is aging. As dementia is one of the top 10 leading causes of death in older adults, there is a need for various services and support that can be utilized by caregivers of persons with dementia (PWD). However, research on service utilization by caregivers of PWD is limited. This study aims to compare the utilization of dementia/Alzheimer's services among Korean, Vietnamese, and non‐Hispanic White caregivers for PWD in the state of California.

**Methods:**

The cross‐sectional analysis included a total of 179 participants from the Dementia Family Caregiver Study, which focuses on building caregiving and stress‐management skills in family caregivers of PWD. Binomial logistic regression was used to estimate the association between race and the utilization of dementia/Alzheimer's services, including in‐home support, adult day care, and support groups. Covariates included caregiver sociodemographics (age, gender, marital status, education, monthly family income, employment status), caregiver characteristics (length of caregiving, instrumental activities of daily living score, receiving help with caregiving, relationship with PWD) ), and PWD‐related factor (health insurance status).

**Results:**

The average age of caregivers for PWD was 66.9 years. Caregivers for PWD were more likely to be female and to have a college or above education for all three racial groups. Compared to non‐Hispanic Whites, Korean and Vietnamese caregivers for PWD were less likely to utilize in‐home support and adult day care services. Korean caregivers for PWD were less likely to attend a support group, while Vietnamese caregivers for PWD were more likely to do so, compared to non‐Hispanic Whites.

**Conclusion:**

Korean and Vietnamese caregivers for PWD demonstrated lower utilization of in‐home support and adult day care services compared to non‐Hispanic Whites. Factors potentially contributing to this disparity may include limited availability of services in languages other than English and a lack of culturally appropriate options. Further research is needed to understand the specific reasons behind the underutilization of these dementia/Alzheimer's care services among these racial groups. Such insights could inform efforts to improve access and enhance cultural competence in service provision.

